# Daily socket comfort in transtibial amputee with a vacuum-assisted suspension system: study protocol of a randomized, multicenter, double-blind multiple N-of-1 trial

**DOI:** 10.1186/s13102-023-00694-4

**Published:** 2023-07-14

**Authors:** Rémi Klotz, Guilhem Emile, Jean-Christophe Daviet, Mathieu De Sèze, Julien Godet, Renaud Urbinelli, Agata Krasny-Pacini

**Affiliations:** 1 La Tour de Gassies Centre for Physical Medicine and Rehabilitation, UGECAM, Rue de la Tour de Gassies, Bruges, 33523 France; 2 Department of Physical and Rehabilitation Medicine, Centre Hospitalier d’Arcachon, Avenue Jean Hameau, 33260 La Teste de Buch, France; 3 Department of Physical and Rehabilitation Medicine, Limoges University, Jean Rebeyrol Hospital, Avenue du Buisson, 87170 Limoges, France; 4 Physical and Rehabilitation Medicine Unit, Bordeaux University Hospital, University of Bordeaux, EA4136 Bordeaux, France; 5 Clinical Research Methods Group, Laboratory of Bioimaging and Pathologies, UMR CNRS 7021, University Hospitals of Strasbourg, Illkirch, Strasbourg, France; 6 Clin-Experts, Tour Rubis, 75013 Paris, France; 7 Department of Physical and Rehabilitation Medicine, UF 4372, CHU de Strasbourg, Institut Universitaire de Réadaptation Clémenceau, 45 Boulevard Clémenceau, Strasbourg, 67000 France; 8 Department of Psychiatry, Hôpital civil, INSERM 1114, Strasbourg University Hospital, Strasbourg, France

**Keywords:** Amputation, Elevated vacuum, Vacuum-assisted suspension, Active vacuum, Prosthetic suspension system, N-of-1 Trial

## Abstract

**Background:**

The main aim of this paper is to present the feasibility of rigorously designed multiple N-of-1 design in prosthetics research. While research of adequate power and high quality is often lacking in rehabilitation, N-of-1 trials can offer a feasible alternative to randomized controlled group trials, both increasing design power at group level and allowing a rigorous, statistically confirmed evaluation of effectiveness at a single patient level. The paper presents a multiple N-of-1 trial protocol, which aim is to evaluate the effectiveness of Unity, a prosthetic add-on suspension system for amputees, on patient-reported comfort during daily activities (main outcome measure), prosthesis wearing time, perception of limb-prosthesis fitting and stump volume and functional walking parameters.

**Methods:**

Multicenter, randomized, prospective, double-blind multiple N-of-1 trial using an introduction/withdrawal design alternating Unity connected/disconnected phases of randomized length on twenty patients with unilateral transtibial amputation. The primary outcome measure is the Prosthetic Socket Comfort Score (SCS), a validated measure of comfort, administered daily by an phone app designed for the study. Secondary outcomes measures will be collected during the 50 days period of the N-of-1 trial: (1) by the same app, daily for patient-reported limb-prosthesis fitting, stump volume variation, and daily wearing time of the prosthesis; (2) by a pedometer for the number of steps per day; (3) by blind assessors in the rehabilitation center during adjustment visits for functional walking parameter (L-Test, 6-minute walk test), and by the patient for the QUEST, and ABC-S. Effectiveness of the Unity system regarding SCS and daily secondary outcome measures will be tested by randomization test. The secondary outcome measures assessed during visits in the rehabilitation center will be analyzed by Non Overlap of All pairs. An estimate of the effect on the amputee population will be generated by aggregating each individual clinical trial (N-of-1 trial) by Hierarchical Bayesian methods.

**Discussion:**

This study protocol was designed to answer the question “which device is best for THIS patient" and to conclude at a group level on the effectiveness of a new devic, using a Multiple N-of-1 trial, which is promising but underused in prosthetics research so far.

**Trial registration:**

N° ID-RCB 2020-A01309-30 Clintrial.gov : NCT04804150 - Retrospectively registered March 20th 2021.

## Keys messages

Multiple N-of-1 trials represent a switch in the rationale behind the choice of a medical device. Instead of selecting prosthetics based on the mean response to the device of a group of participants, multiple N-of-1 trials allow to answer the question “which device is best for THIS patient”, using a rigorous, recognized, scientific methodology allowing individual results as well as group analysis.

This Multiple N-of-1 trials is an approach in an ecological environment and with daily measures that will provide data closer to daily prosthetic use than trials testing device effectiveness outside of daily life;

## Background

Lower limb amputation has a major functional impact and significantly reduces independence in daily life [1]. An adequate socket fit on the residual limb is essential to ensure the patient's comfort but may be impeded by stump volume variations. Movements of the residual limb in the socket may lead to pain, sores, poor prosthesis control, or simply lack of comfort resulting in a limitation of prosthesis use and a loss of mobility [2]. Fifty-seven percent of the amputee population are dissatisfied with the comfort of their prostheses [3, 4] this lack of comfort represents the main complaints in amputees [5].

Vacuum-assisted suspension systems (VASS) (or elevated vacuum systems or active vacuum systems) draw out air and create a negative pressure at the residual limb liner and socket interface. They were originally evaluated by Caspers in 1995 [6] to reduce residual limb volume loss. Benefits of the system found in the literature are many: (1) it increases negative pressure during swing phase for better fitting [7] ; (2) it reduces residual limb volume fluctuations which better protects from skin lesions [8]; (3) it enhances fitting and security during walking [9] ; (4) it optimizes balance (BERG scale) and functional walking parameters (Timed Up and Go Test, 6-minute walk test) [10]; and (5) a recent metanalysis suggests that it improves comfort and quality of life [5].

Although VASS require more maintenance, attention and skills for donning and doffing and may be difficult for older patients with amputation [11].

Prescribers have the choice between different VASS: for the WillowWood LimbLogic system^TM^ and the OttoBock Harmony system^TM^, the vacuum is created using a pump and exhaust valve between a knee sealing sleeve and the liner. The disadvantage is that the sealing sleeve must be worn up to the top of the thigh and therefore limits the knee's range of motion [5], keeps warm and creates hyperpressure on the patella.

On the other hand, Össur Unity^TM^ VASS system relies on a mechanical pump which draws air out from the socket at each step without a sealing sleeve. It uses a Seal-in liner with integrated seals on the middle part of the liner. The vacuum is created between the integrated seals and the distal part of the liner. Gholizadeh et al. evaluated the Unity system and found improved proprioception and comfort [12], but the study relied only subjective feedbacks and did not use a control condition. Other VASS have been assessed only in unecological environment (i.e. not within the natural environment of patient’s daily life), giving little insight into the effectiveness of the system regarding comfort and ecological daily use of the system. Therefore, studies using ecological outcome measures and targeting comfort rather than complications of wearing the prosthesis are needed. Ecological measures target patient’s daily life functioning and preoccupations, in his/her natural context and usual activities.

Trials on prosthetics face a number of challenges: (1) the heterogeneity of the amputee population makes it difficult to obtain comparable groups of sufficient size to detect a statistically significant effect (e.g.: in the 2016 VASS meta-analysis [13] the greatest sample was 18); (2) intra-patient variability due to pain, fatigue or due to artificial (in rehab center) versus ecological activities makes single assessment on a given day or in the rehab center not always representative of the patients’ true functioning; (3) differential response to the prosthetic (i.e. some patients presenting with a great benefit, while others showing no benefit or getting worse), is masked by relying on group means.

Multiple N-of-1 trials are increasingly used in drug trials [14] and may represent a turning point for prosthetic trials as well. Multiple N-of-1 trials are multi-cycle within-patient, randomized, double-blind, cross-over comparisons of an intervention (e.g.: a novel prosthesis) and placebo (or a usual prosthesis) measuring immediate treatment effects and using standardized measures of effect [14]. They provide evidence-based information on individual response to treatment and could be used to optimize the management of each individual patient, or to decide the funding of an additional prosthetic such as VAAS. N-of-1 trials can identify those patients who respond (i.e. show gains) - and those who don’t - to an intervention, for chronic, stable conditions, and are therefore well suited for studying prosthetics in the amputee population. In addition, multiple N-of-1 can be aggregated with high level of power to provide a population estimate of effect, but requiring a fraction of the sample size of the equivalent parallel arm in Randomized Controlled Trials (RCT). This particularity is a chance for research on prosthetics where most studies lack power due to small sample sizes. N-of-1 trials are now recognized as Level 1 Evidence by the Oxford Centre for Evidence-Based Medicine [15]. Table 1 adapted from Nikles et al [14], presents the advantages and necessary conditions for multiple N-of-1 trials.

**Table 1:** Rationale for the methodology

**Conditions to use a multiple N-of-1 trial (adapted from Nikles and Mitchell** [16]**)**
• The patient's condition is stable during the phases with minimal fluctuations over time • The treatment does not change the condition but only treats the symptoms • The intervention has a short wash-out, i.e. its effect disappears when it is removed • The therapeutic effect is quick when the treatment is applied and quickly stops when it is removed, i.e. on/off effect • There is no cumulative effect of the treatment
**Advantages to use a multiple N-of-1 trial methodology**
• Patients lost to follow-up or out of the study can still be analyzed on the phases already completed • Possibility to work with heterogeneous samples for individual interpretation • The 2 arms of the treatments are perfectly matched • All patients test both treatments (no ethical problem) • At group level: statistical significance with smaller sample size • Patient-by-patient analysis takes into account inter-individual variability by analyzing each N-of-1 trial to determine is a patient is a responder, non-responder, or is aggravated • Highlights possible inter-individual variability in the global results • Allows to extract an effect even in presence of large intra-individual variability (e.g.: patient performing variably because of pain, fatigue, unusual activity…) by measuring repeatedly the patient over time.
**Disadvantages to use a multiple N-of-1 trial methodology**
• Only treatments with true on/off effects can be explored and the condition must be stable. Therefore, any prosthetic/orthotic inducing cerebral plasticity (e.g.: thumb opponent splints in children) or requiring a long time for the patient to adapt/learn cannot be explored. This limits the range of prosthetic interventions that can be explored by multiple N-of-1 trials. • The population is smaller and may not be representative of the general population^a^. • Inability to explore statistically predictive factors^b^. • Inability to detect low-prevalence side effects.

^a^However, this problem is not specific to N-of-1 trials. Randomized controlled trials (RCT) claim in theory to provide a generalizable population outcome but they mask variability in response to treatment. Even in the most successful RCT study there are individuals whose behavior is not affected or is worsened by treatment, but these results are drowned out by a group mean.

^b^This is also true for randomized controlled trials (RCT). Power is calculated to demonstrate the effect of a treatment and not to determine predictive factors and responder characteristics.

The aim of the study is to evaluate the effectiveness of the Unity VASS on patient-reported comfort (using the Socket Comfort Score) during daily activities, in ecological environment, in patients with unilateral transtibial amputation blinded to the connection or disconnection of the Unity system, using a randomized, double-blind, multicenter prospective multiple N-of-1 trial. The secondary objectives are to evaluate whether Unity system leads to: (1) increased wearing time with the prosthesis, (2) increased number of steps per day, (3) decreased feeling of stump volume variation; (4) improved perception of limb-prosthesis fitting; (5) increased functional walking parameters.

## Methods & design

This multicentric prospective, randomized, double-blind, multiple N-of-1 trial, will include 20 patients recruited non-concurrently in 4 rehabilitation centers in France, in an 18-months period, representative of the overall French amputee population, in terms of patient’s characteristics and type of amputation performed by the local surgery teams. Because these centers have a large geographical recruitment, patient of all demographic and socioeconomic status will be represented Each patient will be referred by his/her private prosthetist to the inclusion center, which will allow to include patients, who do not have any medical or rehabilitation follow up anymore. Inclusion criteria are presented in Table 2. All concomitant care is allowed, apart from testing another new prosthetic device. Each patient will sign an informed consent, the consent will be collected by each main investigator of the center.

**Table 2:** Inclusion and exclusion criteria

**Inclusion criteria**	**Exclusion criteria**
- Male or female aged >18 years old - Unilateral transtibial amputation > 6 months - Equipped with a Seal-in liner^a^ without VASS since at least 3 months - Equipped with a class II or III prosthetic foot^b^ compatible with the Unity system - ICF classification d4602 and/or d4608^c^ [17] - Presenting some discomfort in the socket evaluated by a SCS ≤ 7/10 - Availability of daily smartphone use and functional 4G connection	- Cognitive impairment impeding comprehension of the trial instructions - Patients who have already been equipped with the Unity VASS system in the past - Pregnancy - Adult under curatorship or guardianship - Patient with no health insurance - Severe comorbidities

*VASS* Vacuum-Assisted Suspension System, *ICF* International Classification Of Functioning, Disability and Health, *SCS* Socket Comfort Score

^a^Seal-in is an Össur liner, compatible with the Unity system

^b^Class II or III stand for ICF d4602 and d4608, respectively, in the French National Health system

^c^d4602 = moving outside the house and other building; d4608 = other specified activities related to moving in and out of other locations (i.e. physical activities, professional activities, etc..)

### Design

After the inclusion visit, a prosthesis (with an Iceross Seal-In™ liner) equipped with an Össur-compatible foot and a Unity system will be manufactured for each included patient. Each center will have one single prosthetist, trained and qualified for the Unity system by the Össur company, who will manufacture all the prosthesis of the trial but will be independent of the team performing the study. Each patient will wear the new prosthesis with the Unity system connected for 28 days, before beginning the N-of-1 trial. This acclimation period is necessary to adjust the prosthesis and obtain a stability in patient’s daily activities.

**Fig. 1:**
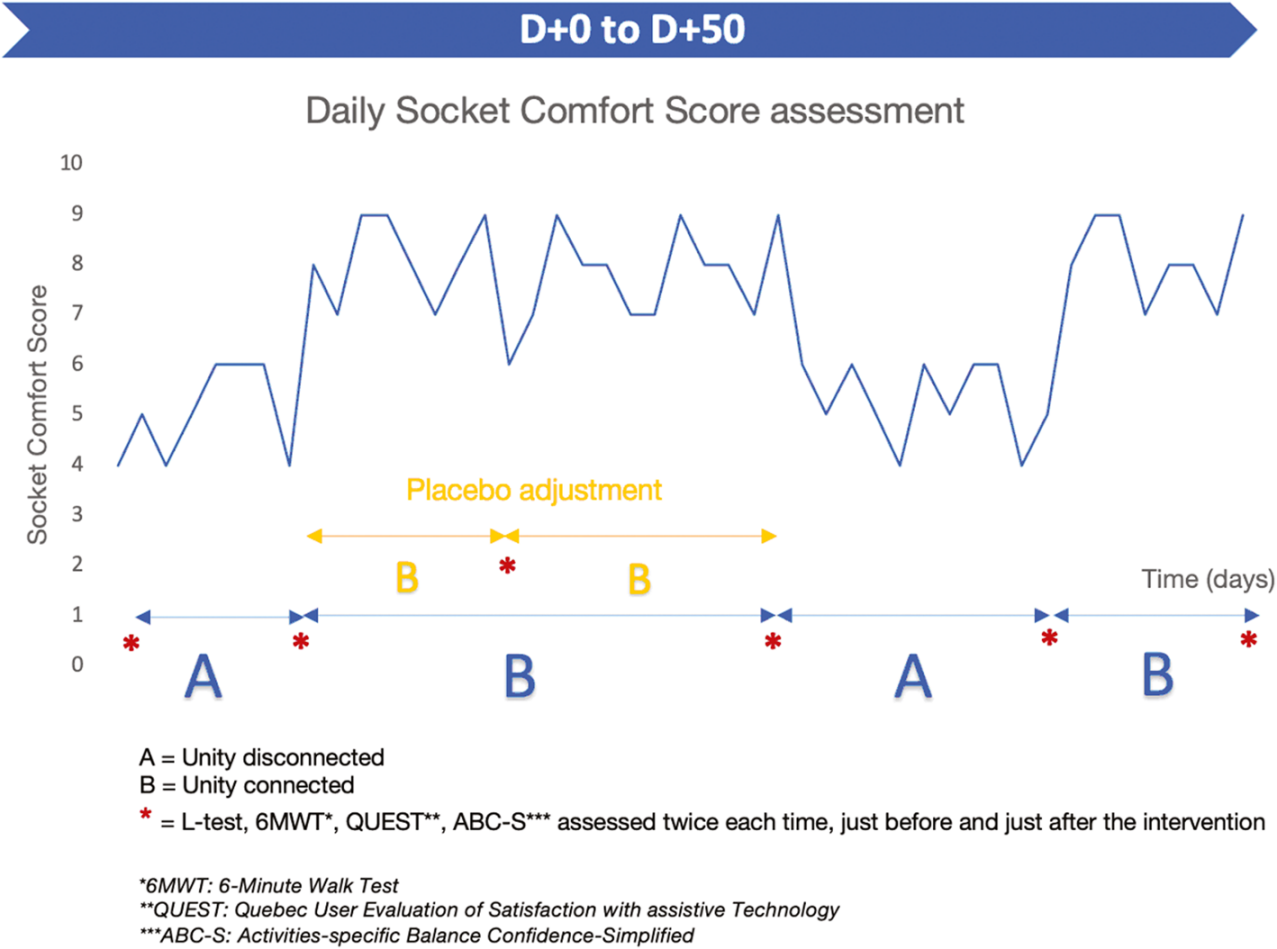
Study Design: hypothetical data explaining the N-of-1 design based on phase length randomization and additional placebo phases change

The N-of-1 trial will then last 50 days for each patient, following a design with random alternation of Unity connection and disconnection of minimum 7 days periods. This minimum period has been decided in order to include a weekend in each period, as patient’s activity level is expected to be variable between weekdays and weekends. Randomization will be performed on R, SCDA (Single Case Data Analysis) plug-in package, by an off-center independent operator. The randomization will use a phase length ABAB randomization (i.e. allowing periods of unequal duration, which allows to interpret results with individual statistical significance) rather than block randomization commonly used in drug trials. “A” is for Unity system disconnected, and “B” for Unity system connected (see Fig. 1). However, because the patient has to come to the rehabilitation center at each phase change which would not allow the patient and the assessors to be blind to the next phase content, phases of a duration >14 days will be further randomized into two half-phases (of 7 days minimum), of the same content i.e. either connected or disconnected. A placebo visit between the two half-phases will allow to keep the blinding. The maximum phase duration obtained by ABAB randomization is 29 days, which will be further randomized to half-phases of minimum 7 days, meaning that the maximum duration of a half-phase is 22 days.

The schedule of each random phase transition from each patient will be transmitted to the health care manager responsible for scheduling patients visits and prosthetist’s activity. Participants, care providers and those assessing outcomes will be blind to the placebo or true phase change. Each phase change will be performed by a different prosthetist from the one who manufactured the prosthesis. At true phase transition, a prosthetist will either connect or disconnect Unity and a third prosthetist verify it after the procedure. S/he will not make any changes if no phase transition is expected (placebo transition). However, s/he will ensure that the prosthesis is properly adjusted. It is not possible for the patient to know whether the Unity tube is connected or not (no visual change).

If the patient misses a phase transition visit, the visit will be rescheduled as close as possible to the planned visit, and the modification of phase length notified. At the end of the trial, patients will be allowed to keep the Unity System if they wish, free of charge. In case of unlikely adverse effects in wearing the prosthesis, the trial will end for that particular patient and s/he will return to his usual prosthesis. This is however very unlikely, as before the trial, the patient will wear the prosthesis for 28 days (which will detect any problems before the trial starts). No emergency unblinding is needed as the prosthetist performing the adjustments is not blind and will notify any adverse effects due to the Unity system.

### Outcome measures

The primary outcome measure is the Prosthetic Socket Comfort Score (SCS), an 11-point numerical scale rated from 0 to 10 [18], based on the question: ‘On a 0 – 10 scale, if 0 represents the most uncomfortable socket fit you can imagine, and 10 represents the most comfortable socket fit, how would you score the comfort of the socket fit of your artificial limb at the moment?’. Test-retest reliability found an intra-class correlation coefficient at 0,79 when the SCS is administered by electronic versions. The minimum detectable change was estimated between 2.73 and 3.26 [19]. The SCS is a validated measure of comfort, commonly used in France but which has never been validated in French; therefore, a French back translation was carried out (appendix 1) to validate the formulation. The SCS will be administered daily by an phone app designed for the study and previously tested for functionality with one patient. It will be recommended to the patient to rate the SCS at a fixed time each day. In case the patient forgets to rate the SCS, s/he will receive a reminder text message between 7pm and 8pm. In case of participant’s low participation in providing their daily SCS, the participant will be phoned by the investigator of the center to increase motivation. Charts of missing data will be updated every month and solution for non-compliant participants discussed with investigators.

Secondary outcomes measures will be collected during the 50 days period of the N-of-1 trial: (1) by the same app, daily for patient-reported limb-prosthesis fitting, stump volume variation, and daily wearing time of the prosthesis (see Table 3); (2) by a pedometer for the number of steps per day; (3) by blind assessors during adjustment visits in the rehabilitation center for the other secondary outcomes listed in Table 3.

**Table 3:** Secondary outcomes

**Daily (ecological)**	**Measurements**
**Pedometer**	Number of steps
**Patients-Reported Outcomes Measures (PROMs)**	
- 2 PROMs about indirect assessment of the stump volume variation:	
** 1.a** “How many times have you removed your prosthesis during the day (not counting the evening’s removal) to add compensation sheaths? “	0, 1, 2, 3, 4, >4,
** 1.b** “Did you have the fear at one (or more) moment(s) of the day, of losing your prosthesis, that it would come off by itself? “	VAS^a^: 0 = not at all 10 = yes a lot
- 2 PROMs on the limb-prosthesis fitting:	
** 2.a** “Do you feel in control of your prosthesis, as if the prosthesis is one with you?”	VAS^a^: 0 = not at all 10 = perfectly
** 2.b** “Do you feel pistoning movement of your prosthesis when walking (sensation of vertical movement of the prosthesis because of the stump when walking)?”	VAS^a^: 0 = not at all 10 = yes a lot
- Wearing time of the prosthesis	In hours
**At each phase change visit twice**^b^	
**Functional walking parameters**	
- L-Test [20]	
- 6-minute walk test [21]	
** Satisfaction and balance questionnaires**	
- Quebec User Evaluation of Satisfaction with assistive Technology (QUEST)^c^ [21, 22]	
- Activities-specific Balance Confidence-Simplified (ABC-S) scale [23]	

^a^*VAS* Visual Analogue Scale

^b^Before and after the prosthetist checks OR connects/disconnects the Unity system

^c^The assessment tool was developed simultaneously in French and English. In French, the assessment is entitled ÉSAT for “Évaluation de la Satisfaction envers une Aide Technique”

Additionally, the app will allow to monitor weekly side effects and factors that may influence the daily ecological outcome measures: patients will answer weekly the two questions: “Have you experienced any unusual event this week that may have affected your prosthesis wearing time, use or comfort (illness, accident, fall, unusual activity, etc.)?” “Have you had an unusual problem with your stump or prosthesis: sweat, wound, unusual pain, abnormal noises from the prosthetic, accidental loosening?”. Responding to all questions has been tested and takes 1 to 3 minutes par day. For patients not familiar with the app, a paper version will be proposed.

For each patient, clinical characteristics will be noted : health status, time of day that data are collected, activity level according to the International Classification of Functioning, Disability and Health (ICF) d4602 and/or d4608 [24], age, and cause of amputation as realized in the study of Sanders et al. [16]. This data will be collected in a paper CRF (with double data entry).

### Sample size calculation

#### Power calculation for each N-of-1 trial (i.e. each patient)

One method for calculating power in a single N-of-1 trial (i.e. for a single patient) is calculating the number of possible randomization assignments [25, 26] which depends on the type of randomization, the number of phases and the number of measurements per phase. The greater the number of possible assignments, the greater the possibility of detecting a treatment effect that exceeds the threshold of significance of 0.05 if an effect exists. With 4 true phase transitions, with a minimum duration phase of 7 days, a maximum study duration of 50 days, and 1 measurement per day, the number of assignments generated by this design is therefore 2300, allowing a significance of up to *p =* 0.0004 for each patient, as calculated on R package for single-cases [17].

#### Number of subjects for the aggregation of the N-of-1 trials

For aggregating the multiple N-of-1 trials, we used the average comfort of a lower limb amputee 7.2/10 (SD= 2.3, inter-patient variance of 5.3) found by Hafner Brian et al. [19] and, (because of lack of data on Unity) our clinical experience that the Unity system would increase comfort by 2/10 points. Based on the calculation proposed by Senn [27] (formula 3 and 4 in the study) to calculate the necessary number of subjects and wanting to detect a clinical difference of 2, with an alpha at 5% and a power at 80%, and assuming that the intra-patient variance is 2, 16.35 subjects are required. If patients stop the study, they can contribute to the analysis of each completed phase. Recruiting 20 patients will allow to satisfy the sample size even in case of early dropouts.

### Data management and independent committee

Each investigator will be responsible for ensuring data completeness. Data management will be handled centrally by the Clin Experts Company. The research in the investigational centers and management of subjects will comply with the Declaration of Helsinki and Good Clinical Practices. Each investigator will be monitored by telephone on a regular basis to keep informed about the progress of the trial, adverse events that occurred during the trial and possible recruitment difficulties. A study progress report will be drawn up every 30 days for the duration of the study. Clin Expert (RU) is responsible for study supervising data collection, auditing recruiting centers for regulatory procedures, for data completeness and supervision of investigators. No steering committee monitoring adverse effects is used because there is no particular risk for the participants (apart from possible lesser comfort). Conversely, an independent committee (JCD, MDS) will check data and protocol integrity. Data statistical interpretation will be performed by a statistician blinded to the hypothesis of the study (JG).

### Ethics committee approval and dissemination of results

This protocol of the study (version 2) was approved by the French Committee for the Protection of Research Participants “CPP SUD MÉDITERRANÉE 1”, (i.e. official French Institutional Review Board) on July 20, 2020 (2020-A01309-30), and by the French Health Products Safety Agency (ANSM) on June 26, 2020 (2020-A01309-30). Prior to the start of the study , the High Authority of Health reviewed the methods, and approved the use of a multiple N-of-1 trail for determining the use (and reimbursement) of Unity. Data will be collected according to methodology (MR-001) issued by the French Data Protection Authority (the, "CNIL"). This methodology is used to simplify the authorization process for the processing of personal data in France and to enforce protection of personal data. Data collected will be collected on paper form (All important protocol modifications will be communicated to both CPP and ANSM, and then transmitted to each center after validation). ANSM and the French High Authority oh Health will be notified of the results and given access to raw data on request. Each participant will receive a personalized statistical analysis report of the effectiveness of the system. A paper of the results will be produced by the same authors, and 3 additional center investigators, without any third party or medical writer involved, apart from English correction.

### Statistical analysis

#### Statistical analysis of each patient’s N-of-1 trial

Effectiveness of the Unity system regarding SCS and daily secondary outcome measures will be tested by randomization test [20, 28], using the mean of each phase. Randomization allows reliable statistical analysis of auto correlated data (such as data from N-of-1 trials) and allows to extract a statistical effect for each participant separately. Missing data points will be managed by multiple imputation method. In case of a rescheduled transition visit, the real transition date and not the initially scheduled one will be used.

The secondary outcome measures assessed during visits in the rehabilitation center will be analyzed by NAP (Non Overlap of All pairs) [21, 22]. NAP is an estimate of the probability that a randomly selected point in Phase B shows an improvement over a randomly selected point in Phase A with θ=Pr(YB>YA)+0.5×Pr(YB=YA). Since NAP's interpretation of effect size according to Cohen's rules (d=3.464⁎(1√(1-NAP)/.5) has been shown to be inadequate, Parker's criteria will be used [21] (effect present but small if NA*P =* 0.5-.65; moderate effect: .66-.92; large effect: .93-1.0.). NAP [21] will also be applied to daily outcome measures, in addition to randomization tests, to obtain an effect sizes.

#### Aggregation of the 20 N-of-1 trials

Data from all participants will be synthesized visually by Brinley Plots [29]. The clinical characteristics of responders and non-responders to the Unity system will be compared by non-parametric methods (Fisher's exact test and Wilcoxon's test). An estimate of the effect on the amputee population will be generated by aggregating each individual clinical trial (N-of-1 trial). Hierarchical Bayesian methods based on normal likelihood distributions [30] or non-normal likehood distributions [31] will be used. We will associate a non-informative prior distribution (Jeffreys' prior). By combining prior distributions and likelihood distributions, these methods provide probabilistic estimates of individual and group effects. Thus, the results of all completed phases will be combined to produce a posterior probability of the overall difference between Unity connected phases and Unity disconnected phases. The Unity system will be judged to be effective on the whole population if the highest density interval at 95% (HDI 95%) of the posterior probability of the mean difference favouring the treatment exceeds 0.975. It will be considered ineffective if the posterior probability of the average difference in favour of the treatment is below 0.025.

Data analysis will be conducted by a statistician blinded to phase allocation, using ABAB randomization tests with the ExPRT macro package (*Excel*^®^ *Package of Randomization Tests*) Statistical Analyses of Single-Case Intervention Data (Version 3.2, October 2018) developed by B.S. Gafurov and J.R. Levin [23], R (version 3.6) for Bayesian statistics and NAP calculator (https://jepusto.shinyapps.io/SCD-effect-sizes/).

## Discussion

This study brings innovative elements in assessing ecological comfort and using multiple N-of-1 trial methodology in the prosthetic field. Previous research suggested that VASS improved slightly gait parameters, but none assessed comfort as the main outcome, and not ecologically as we intend to. Further, most research focuses on limiting prosthetic-related problems (e.g.: sores, volume loss), or walking parameters, while the main patient’s priority, after these are sufficiently addressed, is comfort. To adequately use multiple N-of-1 trials, a true on/off effect is needed, which connecting/disconnecting Unity allows perfectly. Double-blinded randomized trials are rarely feasible in the prosthetic field, which is another strength in this project. Finally, having a single prosthetist to manufacture all the devices in a given center, should reduce inter-patient variability due to technical issues, and concentrate the N-of-1 trial on inter-individual differences due to the Unity system being tested.

Multiple N-of-1 trials represent a switch in the rationale behind the choice of a medical device: instead of selecting prosthetics based on the mean response to the device, multiple N-of-1 trial allows to answer the question “which device is best for THIS patient”, using a rigorous, recognized scientific methodology. Moreover, it allows to obtain individual results, as well as group results, with greater power than usual RCTs (often including insufficient samples of amputees). Using PROMs (and especially comfort) and ecological repeated measures, the results obtained by this trial are more likely to correspond to the true effectiveness of the prosthesis in daily life and in patients’ personal activities. Double blind multiple N-of-1 trials could be the methodology that adds objectivity to patient’s usually subjective choice of a prosthesis, increasing therefore science contribution above marketing contribution in this highly competitive field. Extending this rationale to decisions of funding of expensive devices, a future health policy could be to fund a costly device only for patients showing a statistically significant improvements based on a N-of-1 trial, rather than using general funding criteria. Using PROMs can be a strength as they give insight into patient’s subjective appreciation of the prosthesis, but PROMs may be very biased if they relate to costly devices without blinding to the condition. Here, we hope that blinding will allow PROMs to be less biased. Thus, showing objectively a benefit could allow many people with transtibial amputation to upgrade their comfort in their daily life.

Some limitations must be highlighted. Firstly, the funder of this study is the Össur company, which has created and sells the Unity system. However, the company does not participate in the interpretation of data or in drafting this manuscript. A result report will be prepared for the High Authority of Health (organization that decides the types of prosthetics funded by the health system in clinical care in France) but publishing the protocol prior to the trial will ensure that the methodology is not changed or results not interpreted in a biased way. Secondly the order of phases in N-of-1 trials should be completely random, while here all patients begin the study with a Unity disconnected phase (after an acclimation time with the Unity connected). This was decided because a ceiling effect (high SCS) was expected even without Unity connection i.e. before knowing that a better comfort is possible, patients are likely to rate SCS as very high, (as long as they have no sores or obvious discomfort). This phase order was expected to educate the patients to be aware of their comfort, but is criticizable, as it may induce lower rating of SCS after Unity’s first disconnection. Finally, usual randomization in N-of-1 trial are block randomizations with equal phases length. Phase length randomization (giving unequal phase lengths) comes from behavioral research and is less used in medicine. Here the latter was preferred, in order to obtain a statistical significance and a power calculation for each N-of-1 trial separately.

## Data Availability

The datasets generated and/or analysed during the current study are not publicly available due to third party (Össur France) restrictions but might be available from the corresponding author on reasonable request.

## Abbreviations

PROMs: Patients-Reported Outcomes Measures;
RCT: Randomized Controlled Trial;
SCS: Socket Comfort Score (or Socket Prosthetic Comfort Score);
VASS: Vacuum-Assisted Suspension System
